# Intratumoral injections of G100 (synthetic TLR4 agonist) increase trafficking of lentiviral vector-induced antigen-specific CD8 T cells to the tumor microenvironment

**DOI:** 10.1186/2051-1426-3-S2-P290

**Published:** 2015-11-04

**Authors:** Tina C Albershardt, Andrea J Parsons, Peter Berglund, Jan ter Meulen

**Affiliations:** 1 Immune Design, Seattle, WA, USA

## 

The clinical efficacy of tumor-specific effector T cells can be limited by their proper trafficking to the site of the tumor and the immunosuppressed local environment. Strategies to improve homing of effector cells to tumors and to enhance activity of these effector cells could further unlock the potential of active cancer immunotherapy. G100 is the synthetic TLR4 agonist glucopyranosyl lipid adjuvant (GLA) formulated with an oil-in-water stable emulsion and has been shown to induce T cell homing chemokines CXCL9 and CXCL10. We assessed here whether intratumoral injections of G100 could improve trafficking of tumor antigen-specific CD8 T cells to the tumor microenvironment (TME), thereby achieving better anti-tumor control.

Untreated, B16F10-OVA tumor-bearing mice generated no detectable levels of ovalbumin (OVA)-specific CD8 T cell response as assessed by flow cytometry analysis. In contrast, tumor-bearing mice immunized with ZVex^TM^/OVA, a novel lentiviral vector platform expressing OVA, generated 8-9% tumor antigen-specific effector and memory CD8 T cells within the peripheral tissue, which remained detectable at low levels even up to 35 days post-immunization. Tumor-infiltrating lymphocytes (TILs) isolated from mice treated with ZVex/OVA alone had an average of 16.6% antigen-specific CD8 T cells, whereas those from mice treated with ZVex/OVA and G100 had 25.9%. While ZVex/OVA-induced antigen-specific CD8 T cells infiltrated the tumor without G100, most of these CD8 T cells did not remain in the TME over time. Intratumoral injections of G100 not only increased the total number of effector CD8 T cells within the TME but also kept the CD8 T cells within the TME over time. Furthermore, tumor-bearing mice treated with ZVex/OVA and G100 had significantly improved survival with slower growing tumors.

We show here that intratumoral injections of a formulated synthetic TLR4 agonist, G100, improved vector-induced therapeutic efficacy by increasing trafficking of vector-induced effector T cells toward the TME. Because G100 also stimulates antigen presentation and maturation of dendritic cells, intratumoral G100 following vector-induced generation of antigen-specific CD8 T cells or adoptive transfer of CAR or TCR T cells may be an effective way to increase the therapeutic efficacy of cancer immunotherapy.

